# Exploring the hidden depth by confocal Raman experiments with variable objective aperture and magnification

**DOI:** 10.1007/s00216-021-03678-w

**Published:** 2021-10-01

**Authors:** Barbara Boldrini, Edwin Ostertag, Karsten Rebner, Dieter Oelkrug

**Affiliations:** 1 Process Analysis and Technology, Reutlingen Research Institute, Reutlingen University, Alteburgstr. 150, 72762 Reutlingen, Germany; 2 Institute of Physical and Theoretical Chemistry, University of Tübingen, Auf der Morgenstelle 18, 72076 Tübingen, Germany

**Keywords:** Confocal Raman micro-spectroscopy, Raman depth profiling, Thickness determination, Refractive index mismatch, Composite layers, Polymer films

## Abstract

The article analyzes experimentally and theoretically the influence of microscope parameters on the pinhole-assisted Raman depth profiles in uniform and composite refractive media. The main objective is the reliable mapping of deep sample regions. The easiest to interpret results are found with low magnification, low aperture, and small pinholes. Here, the intensities and shapes of the Raman signals are independent of the location of the emitter relative to the sample surface. Theoretically, the results can be well described with a simple analytical equation containing the axial depth resolution of the microscope and the position of the emitter. The lower determinable object size is limited to 2–4 μm. If sub-micrometer resolution is desired, high magnification, mostly combined with high aperture, becomes necessary. The signal intensities and shapes depend now in refractive media on the position relative to the sample surface. This aspect is investigated on a number of uniform and stacked polymer layers, 2–160 μm thick, with the best available transparency. The experimental depth profiles are numerically fitted with excellent accuracy by inserting a Gaussian excitation beam of variable waist and fill fraction through the focusing lens area, and by treating the Raman emission with geometric optics as spontaneous isotropic process through the lens and the variable pinhole, respectively. The intersectional area of these two solid angles yields the leading factor in understanding confocal (pinhole-assisted) Raman depth profiles.

**Graphical abstract:**

**Figure:**
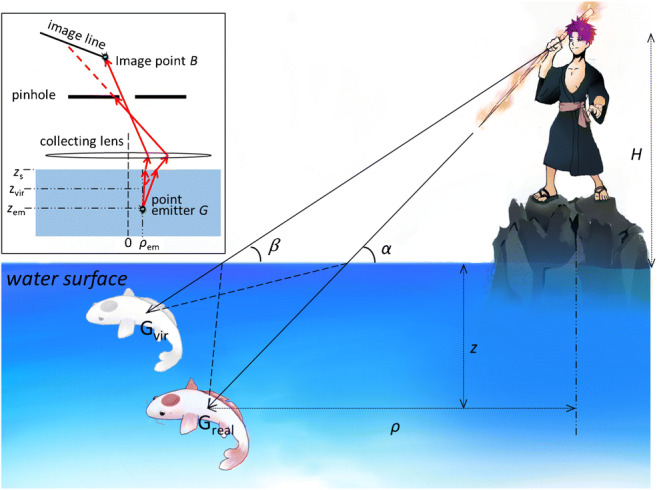
Spearfishing is a well-known example of the effects of refraction at the boundary between two index-mismatched media. The object G_real_ is seen, due to refraction, as G_vir_ from the angle *β* (without knowing the depth position). The real position is obtained under the angle *α*. In a microscope (see inset), index mismatch deforms the image point of G_real_ into an image line. The pinhole substantially reduces deformations and allows the determination of the position of the point emitter G. (Cartoon designed by Sofia Anker)

## Introduction

Scanning Raman micro-spectroscopy is an elegant tool for the chemical characterization and localization of small objects, especially when they are distributed in two dimensions on a planar substrate [1–3]. The lateral resolution can reach values in the < 100-nm scale by means of near-field techniques such as tip enhancement [4, 5]. Diffraction-limited microscopy achieves in the best case a lateral resolution of about 200 nm [6, 7].

It is a challenge to transfer the two-dimensional resolution into the volume of three-dimensional samples. In the axial direction, the spatial resolution is limited with conventional microscopy in the visible region to 2*w*_Z_ ≈ 600 nm (FWHM) [8]. Up to this limit, the thickness of objects can directly be determined with high accuracy from the intensity of the Raman signal [9, 10]. This method is also applicable at the expense of depth resolution to thicker layers, but at some stage the signal height begins to saturate, even for quasi-collimated irradiation [11]. There are many reasons for saturation like aperture, magnification or pinhole size on the microscope side, thickness, depth inhomogeneity, surface refraction and total internal reflection, surface roughness, bulk absorption, or elastic scattering on the sample side. The saturation level of the signal height depends on the position of the microscope focus relative to the sample surface. Therefore, a depth scan of the sample can deliver, among others, the axial resolution of the setup, the thickness of the complete sample, and the positions of its components, as well as the angular and radial intensity distribution of the irradiation source. These parameters are covered in this article investigating polymer samples with coplanar smooth phase boundaries and negligible elastic scattering power. Despite these ideal properties, the saturation level falls off, like in many examples of the literature [12–28] with the in-focal depth position due to the aberration of confocality as a consequence of refractive index mismatch (RIM). The article therefore aims to reduce the drop of intensity as much as possible so that even deeply located objects can be correctly analyzed in terms of chemical composition, size, and shape. The experimental results are accompanied by theoretical calculations based on literature work on electromagnetic diffraction [20–27], Gaussian beam [26, 28], and geometric optics approaches [29, 30]. The level of calculation which is necessary to reliably reproduce the experiments depends on the sample thickness and the microscope objective. We will show that deep layer regions of thickness ≥ 10 μm can be correctly assigned with a single formal equation containing only the effective depth resolution of a small aperture objective, whereas large apertures require more advanced theoretical effort taking into account a finite-sized irradiation waist, the width of the laser beam cross section through the irradiation lens, and the pinhole diameter as variables. Our numerical evaluation methods are in good agreement with the experiments, with the side effect that unexpected discrepancies can be assigned to irregularities of the sample, like surface roughness or elastic bulk scattering. These properties will be described in a follow-up contribution.

## Materials and methods

### Materials

Experiments are carried out on the following materials: a crystalline silicon disk, provided by WITec GmbH; a commercially available planar transparent polystyrene sheet; a glycerol/water mixture (86/14, w/w); a polystyrene rigid foam sheet with macroscopically turbid appearance; a standard colorless transparent adhesive tape; and a multilayer polymer film for food packaging, provided by Peter Ludwig from K-Pack Folien GmbH. Table 1 lists some of the geometric and optical properties of the materials used in this study.

**Table 1:** Geometric and optical properties of the materials used in this study

**Material**	**Layer thickness*d***_**real**_**, μm**	**Refractive index*n***_**D**_^**20**^	**Characteristic Raman shiftΔ*ν*(cm**^**−1**^**)**	**Assignment**
**Silicon disk**	~800	3.96	520	Si-Si
**Polystyrene**				
(a) Transparent layer	124	1.59	1001	Benzene ring breathing mode
(b) Rigid foam [11]	1400	1.59	1001	
**Glycerol/water (86/14, w/w)**	→∞	1.45	1450	δ CH_2_
**Adhesive tape, consisting of:**				
Polypropylene backbone (PP) Adhesive acrylic resin (PMA)	Total 40	1.49 1.48 [31]	399 1730	ω CH_2_ & δ CH [32] ν C=O
**Packaging film, stack of**				
Polyamide Polyethylene Poly-ethylene-vinyl alcohol (EVOH) Polyethylene	Total 72	1.53 1.51 1.53 1.51	1623 2860 825 sh, 850 2860	Amide I ν CH γ_r_ CH_2_, ν CC [33] ν CH

### Confocal Raman measurements

A confocal Raman microscope alpha300 SR from WITec GmbH is used for the acquisition of the Raman depth profiles. Line maps in the axial direction are collected using a precision motorized stage with a *z*-axis controller including a piezo table. The best axial resolution is 0.1 μm within the range of the piezo table. The specimens are irradiated for Raman excitation by a frequency doubled Nd:YAG laser with wavelength *λ* = 532 nm. The radiation is transferred to the microscope via a single-mode optical fiber with a 3.5-μm core diameter. The laser beam is first expanded and then collimated and hits as parallel beam the rear side of the objective and further irradiates the sample.

Raman depth scans are carried out with variable angular aperture. For this purpose, we use three Carl Zeiss objectives with different apertures and magnification factors, as detailed in Table 2.

**Table 2:** Specific parameters of the Carl Zeiss objectives

**Objective**	**EC Epiplan** **20×/0.4**	**EC Epiplan** **50×/0.7**	**EC Epiplan-Neofluar** **100×/0.9**
**Magnification factor,*M***	20×	50×	100×
**Numerical aperture, NA**	0.4	0.7	0.9
**Front lens (sample side) diameter (mm)**	4.0	3.1	1.6
**Rear lens (tube side) diameter (mm)**	6.5	6.1	4.1
**Working distance (mm)**	3.1	1.1	0.31

The Raman radiation emanating from the samples is collected by the objectives and then coupled into a multimode optical fiber with a core diameter of *D* = 25 μm, acting as detection pinhole. Subsequently, the Raman signal is spectrally analyzed with a UHTS 300 spectrometer. The Raman spectrum is recorded from Δ*ν* = 0–3600 cm^−1^ with a spectral resolution of 2 cm^−1^.

The depth profiles have been acquired in a single run, using a motorized sample stage for thick samples (∆*z* = 0.25 – 1 μm, maximal scan range −100 to +100 μm) and a high-resolution piezo-driven scan stage for thin samples (∆*z* = 0.1–0.25 μm, maximal scan range −10 to +10 μm).

### Extraction of the experimental depth profiles

For Raman depth profiling, we select Raman bands which are specific for the material. In the case of multilayer samples, the selected Raman bands do not overlap with each other. Subsequently, the depth profiles are obtained by numerical integration of the characteristic Raman band intensities at distinct depths, after background subtraction of the spectra. An overview on the characteristic bands used for the extraction of the depth profiles is given in Table 1.

### Modeling

The double-objective optics of the confocal Raman microscope is modeled here by a single collecting lens (radius *R*_L_, focal length *z*_F_) combined with a circular pinhole (diameter *D*) mounted at variable positions *z*_P_ on the optical *z*-axis (*x*,*y* = 0) of the lens. On the sample side, the pinhole forms an image at *z*_0_ with radius *w*_P_ that depends on *z*_P_, *z*_F_, and *D*. The width of *w*_P_ includes diffraction broadening. The radiation source for Raman excitation is built into the microscope and imaged confocally at *z*_0_ to the circular area π*w*_0_^2^ centered within the pinhole image.

**Fig. 1:**
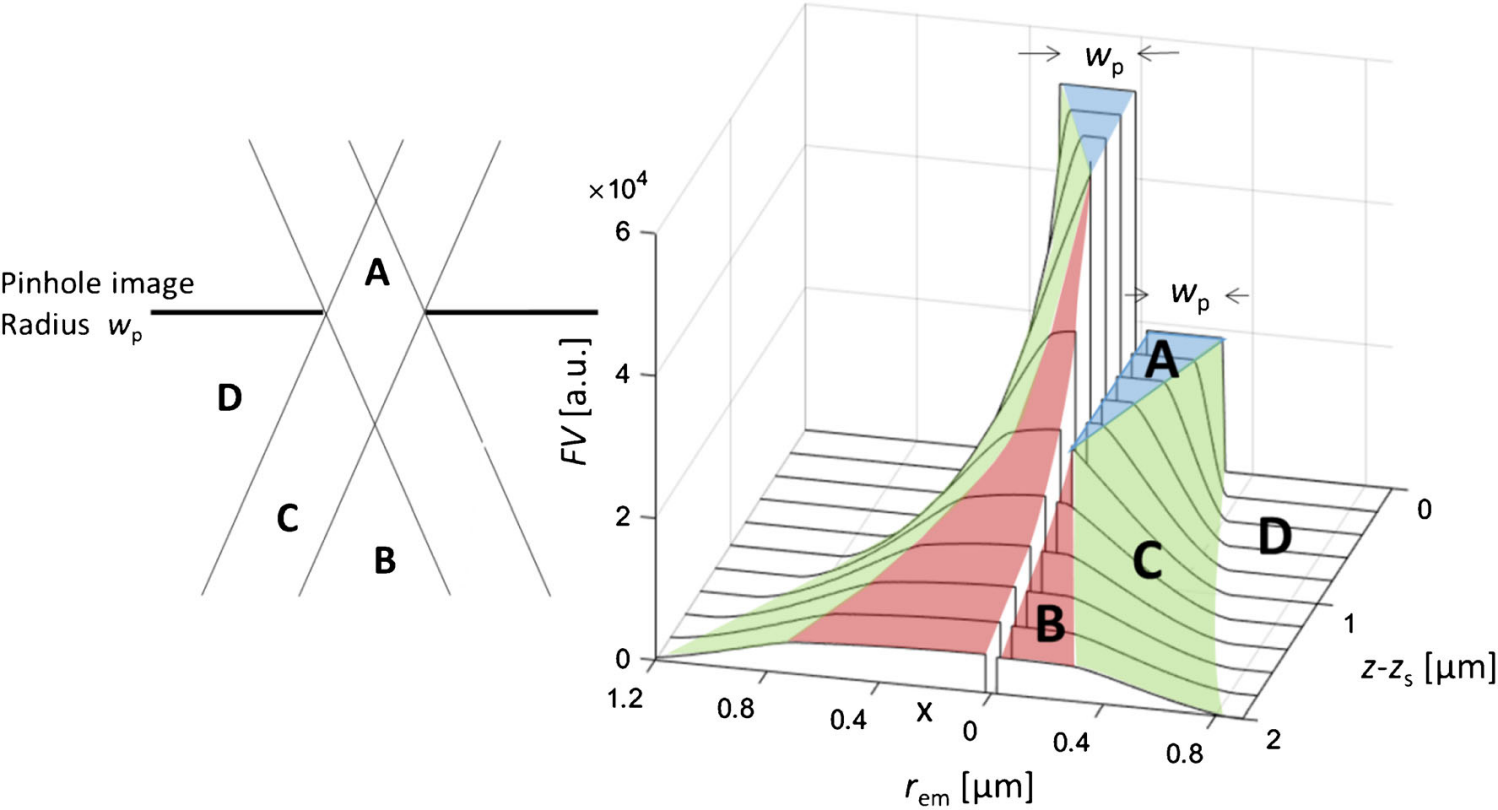
Field of vision (FV) as function of the position of isotropically emitting points G(*r*_em_, *z*-*z*_S_) inside the layer. Pinhole and pinhole image radius *w*_P_ = 0.3 μm (1:1 magnification), *z*_P_ = *z*_0_ = 2*z*_F_ = 1000 μm. Lens radius *R*_L_ = 1000 μm. Left: index match, *n* = 1. Right: index mismatch, *n* = 1.6. Pinhole projection at the surface (*z*_0_ = *z*_S_). Colored areas: angular boundaries of the field of vision

Basic quantities for the analysis of the expected Raman signals are the *Field of Vision* (*FV*, *area*) and the *Field of EXcitation* (*FX*, *number of photons area*^*−1*^*time*^*−1*^). The product of these two quantities forms the *Field of detection* (*FD*). The backscattered Raman signal *X*_R_ is obtained by summing up all contributions to *FD* over the sample volume. Upon conservation of cylindrical symmetry, i.e., perfect alignment of the laser with the pinhole image, one obtains

1 $$ {X}_R\sim \int FDdV=\int FV\ast FX\ 2\uppi r\  dr\  dz $$

where *r* = (*x*^2^ + *y*^2^)^1/2^ is the radial distance from the optical axis, and *dz* ranges over the sample thickness *d.* The *field of vision* describes the detection probability density of every potentially emitting point G(*r,z*) in the sample volume. In this contribution, traditional geometric optics for spontaneous, i.e., incoherent emission, is applied with the additional assumption that G emits isotropic. In the case of refractive index match, the local FV(*r,z*) is proportional to the intersectional area of two solid angles *Ω*_L_ × *Ω*_P_ with G at the apex, one through the lens (*Ω*_L_) and one through the pinhole and the pinhole image (*Ω*_P_), respectively. The results of the model are summarized in Fig. 1 as 3D projections of FV in the vicinity of the pinhole image. The left half stands for the refractive index *n* = 1, the right half for *n* = 1.6. In the latter case, the pinhole image is localized either in front of or directly at the refractive sample surface *z*_S_ = *z*_0_ (pre-focal range). The results are separated into four partial regions A–D.

*Region A* yields the highest detection probabilities for the emitters. Since *Ω*_L_ < *Ω*_P_ and *w*_P_ << *z*, the FV is proportional to *Ω*_L_ and in practice independent of the local position in A

2 $$ FV\sim {\varOmega}_{\mathrm{L}}=4\uppi {\sin}^2\left({\theta}_{\mathrm{L}}/4\right) $$

where *θ*_L_ is the aperture of the lens, *θ*_L_ = 2 arctan(*R*_L_/*z*).

*Region B* forms a cone with the aperture of the lens (for *n* = 1). Since now *Ω*_P_ < *Ω*_L_, the FV is proportional to *Ω*_P_ which intersects fully with *Ω*_L_ but depends strongly on the local coordinates *r* and |*z* -*z*_0_| of G.

3 $$ {\theta}_{\mathrm{P}}=\arctan \left(\frac{w_{\mathrm{P}}+r}{z-{z}_0}\right)+\arctan \left(\frac{w_{\mathrm{P}}-r}{z-{z}_0}\right) $$

*Region C* forms a cone mantle of radial width 2*w*_P_ around region B. The FV is still proportional to *Ω*_P_. However, the intersection with *Ω*_L_ changes from *complete* at the BC-boundary with a sigmoidal decline [34, 35] to *zero* at the CD-boundary (half-shadow region).

*Region D* acts in transparent layers as sink for Raman excitation with no chance to be detected. In turbid systems, region D additionally acts as a potential source of Raman radiation via backscattering into A, B, or C (details in a follow-up publication).

#### The influence of refractive index mismatch

The model sample forms a microscopically planar phase boundary at *z*_S_. We distinguish two positions relative to *z*_0_.

- The *pre-focal range z*_S_ ≥ *z*_0_. According to Snell’s law, the apertures of all point emitters in A are reduced to sin(*θ*_A_) = sin(*θ*_L_)/*n*. The A-field of vision is therefore reduced to FV_A_(*n*) = FV_A_(1)/*n*^2^ (see Eq. (2)). On the other hand, the A-field expands more into the *z*-direction. The apertures of the point emitters in B or C do not change with *n* (compare the amplitudes in the deep *z*-regions of the left and right halves in Fig. 1), but the aperture of the whole cone (B + C) shrinks according to Snell’s law, so that the integral FV_B.C_ reduces in analogy to FV_A_ with *n*^2^. Experimentally, the FV is not “seen” from the real depth position of G, but from the virtual coordinate *z*_vir_, according to

4 $$ \frac{z_{\mathrm{vir}}-{z}_s}{z_{\mathrm{em}}-{z}_s}=\frac{\tan \left({\varphi}_{\mathrm{int}}\right)}{\tan \left({\varphi}_{\mathrm{ext}}\right)}\kern0.5em \overset{\underset{\varphi \to 0}{\lim }}{\to }=\frac{\sin \left({\varphi}_{\mathrm{int}}\right)}{\sin \left({\varphi}_{\mathrm{ext}}\right)}={n}^{-1} $$

where *φ* are the polar angles of radiation inside and outside the layer, respectively. Hence, the real thickness *d*_real_ of a layer is optically compressed to *d*_exp_ by a factor of *n* or more. This effect partly compensates the *n*^−2^ dependence of the detected solid angles so that the total FV as well as the backscattered Raman signal becomes approximately inversely proportional to *n*.

(b) *The in-focal range z*_S_ *< z*_0_*.* Optical refraction at the phase boundary shifts the (*z*_0_-*z*_S_)-position of the pinhole image to deeper *z*-values, for paraxial beams by a factor of *n* at least, for large polar beam angles *φ* considerably more. Hence, the pinhole image of a wide-angle objective becomes misaligned with the consequence of an extended field of vision, especially into the direction of the +*z*-hemisphere. Calculations can be carried out similar to the index-matched situation. However, the distance of the pinhole image against G depends now on *φ*, so that the effective solid angle *Ω*_P_ has to be determined as integral over *d*(cos*φ*).

**Fig. 2:**
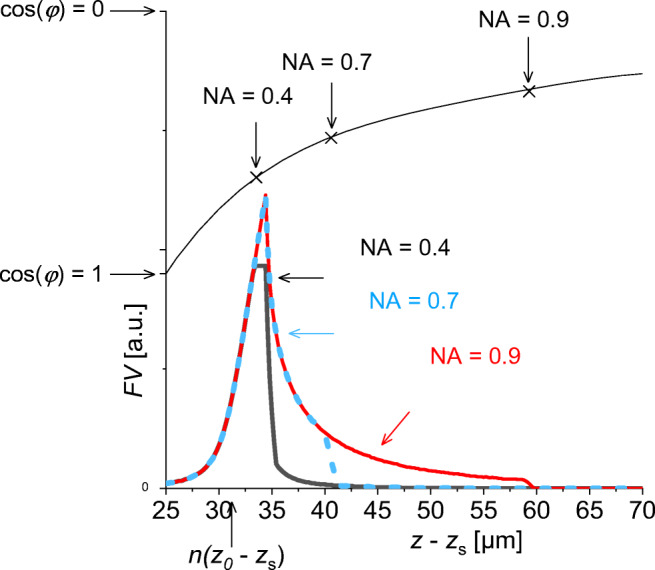
*z*-profiles of the FV, index mismatch *n* = 1.6, for emitting points G(0, *z*-*z*_s_) on the optical axis of the microscope. Compared to the arrangement of Fig. 1, the sample surface is shifted by *z*_0_-*z*_s_ = 20 μm towards the lens, and the lens radius *R*_L_ is introduced as variable corresponding to NA = 0.4 (black line), NA = 0.7 (blue line), NA = 0.9 (red line). Black curve on top: effective pinhole image position as function of the emission angle cos(*φ*) (scaling on the ordinate). The crosses indicate the maximum acceptance angle of emission for different NAs. The extreme values for emission angle 0 and π/2 are shown on the ordinate scale

Figure 2 shows FV curves for emitters G(0, *z*) located at the optical axis in a medium with *n* = 1.6. The pinhole image for *n* = 1 lies at *z*_0_-*z*_S_ = 20 μm below the sample surface. At low *z*-positions of G, FV is formed from paraxial beams only, independent of the NA of the lens. When approaching the effective pinhole image position, also wider angles add to the signal strength. The trace with NA = 0.4 reaches a short plateau as in Fig. 1. Then, the signal decreases again with increasing distance from the pinhole image. Wide angles with their deeper *z*-positions of the pinhole image are truncated by the limited aperture of the objective. The objectives with NA = 0.7 and especially NA = 0.9 accept also deeper *z*-positions before truncation, but only wide angles of emission (for small angles, the pinhole image is too far away). Consequently, the signal of a deeply localized isotropic emitter will be only partly detected. This effect becomes more and more important for increasing *z*_0_-*z*_S_ distances.

**Fig. 3:**
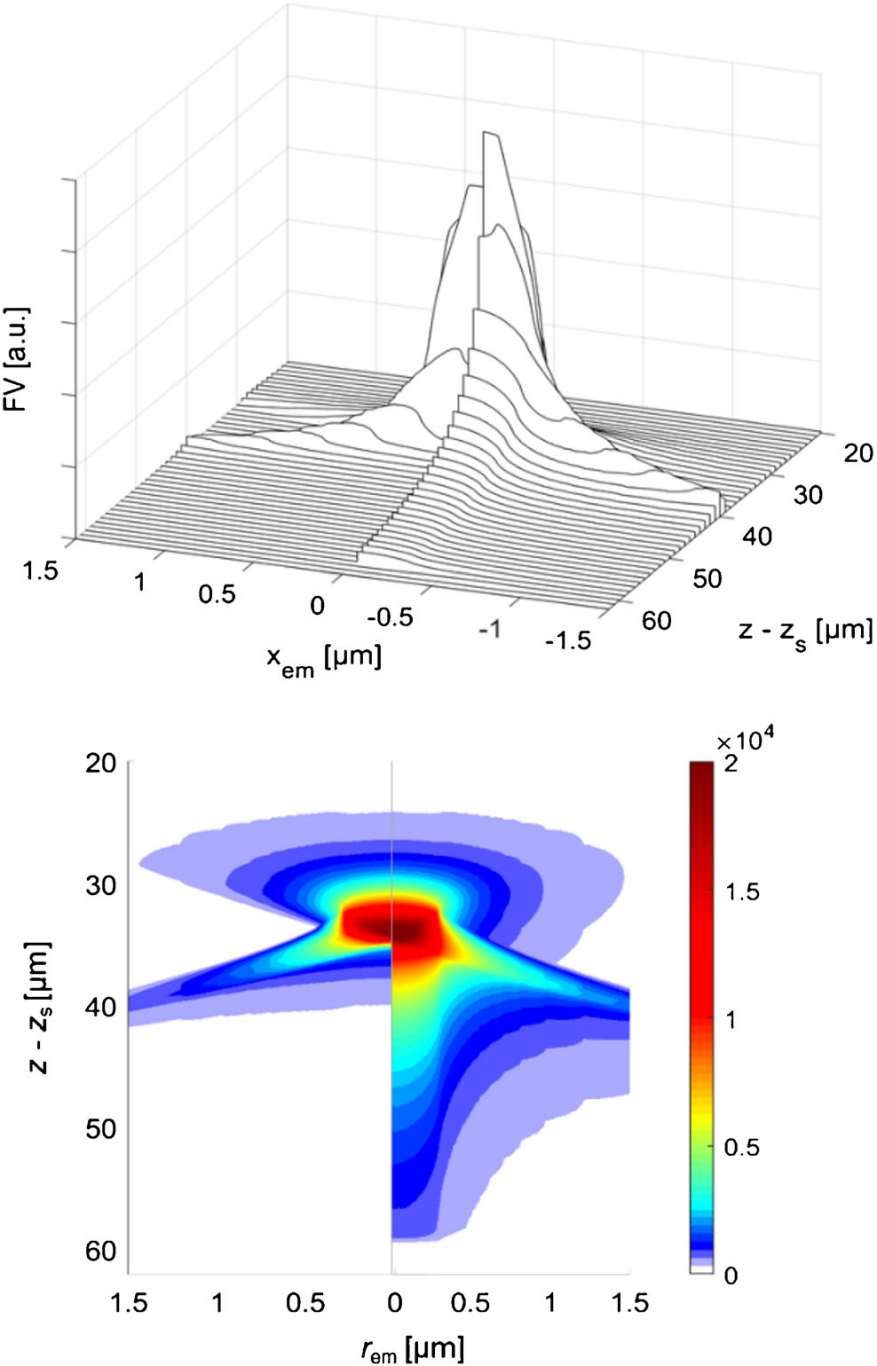
*z*-profiles of the FV, as function of the position of an isotropically emitting point G(*r*_em_, *z*-*z*_s_). Detection geometry as in Fig. 2. with the additional off-axis variable *r*_em_ as distance of G from the optical axis. Left: NA = 0.4; Right: NA = 0.9. Top: representation as waterfall diagram. Bottom: color-coded intensity of FV

Figure 3 describes the FV of point emitters with arbitrary distance *r* from the central axis, split into the calculated results for NA = 0.4 (left half) and NA = 0.9 (right half), both for index mismatch *n* = 1.6. The main differences between the two apertures are found into the paraxial +*z*-direction, where the FV with NA = 0.9 extends by +30 μm beyond the depth of the vision maximum, whereas NA = 0.4 drops down after +4 μm already. The extension differences become much smaller in the sideway +*z*-vision branch, and the differences vanish in the hemisphere directed to the irradiated surface. The latter region is masked in the 3D projection (Fig. 3, top) but becomes clearly visible in the contour plot (Fig. 3, bottom). In total, the FV drops strongly with the *r*-distance from the optical axis. The Raman signal, however, increases linearly with *r*, see Eq. (1), so that the FV regions at small Δ*r*, but not at exactly *r* = 0 are the most important contributors to *X*_R_. This fact was neglected in early publications but was clearly pointed out by e.g. Maruyama et al. [21] who depicted contour plots of FV as calculated for NA = 0.9 with scalar wave optics. Their results are more detailed than ours, but in essence equal to the geometric approximation in Fig. 3.

The *Field of EXcitation* is provided in the present work by a coherent laser source with Gaussian TEM_00_-profile:

5 $$ FX\sim {w}^{-2}\exp \left(-2{\left(\raisebox{1ex}{$r$}\!\left/ \!\raisebox{-1ex}{$w$}\right.\right)}^2\right) $$

First, the laser beam is expanded to fill an appreciable fraction *ff* of the rear objective lens area. Then, the beam is concentrated through the objective to the *z*-dependent width

6 $$ w(z)=\sqrt{w_0^2+{\left( ff\ \frac{R_{\mathrm{L}}}{z_0}\left(z-{z}_0\right)\right)}^2} $$

where *w*_0_ is the smallest spot radius that is obtainable at *z*_0_ for a given magnification and aperture of the objective. The weighted angular distribution of irradiation is given for large distances from *z*_0_ by

7 $$ \tan \varphi =\frac{R_{\mathrm{L}}}{z_0}\exp \left(-2{\left(\raisebox{1ex}{$r$}\!\left/ \!\raisebox{-1ex}{$ ff{R}_{\mathrm{L}}$}\right.\right)}^2\right) $$

Close to *z*_0_, all angles change to normal incidence. We insert tan *φ*_ex_(*r*, *z*_Ray_) = 0 for |*z* - *z*_0_| ≤ *w*_0_.

The calculation of backscattered Raman radiation starts with an ultra-thin layer (*d* ➔ 0) located at *z* = *z*_0_. The detection probability is given by Eq. (1). Then, the layer is moved into ± *z*-direction. This procedure creates wider radial Gaussian distributions of excitation with equal integral flux but lower FV of Raman emission. Mathematically, the detection probability follows a Cauchy-Lorentz distribution. The latter is normalized here to the distribution maximum (not to the area) yielding for the *z*-dependent backscattered Raman signal *X*_R_ the proportionality

8 $$ {X}_{\mathrm{R}}(z)\sim \kern0.5em \frac{w_{\mathrm{z}}^2}{{\left(z-{z}_0\right)}^2+{w}_{\mathrm{z}}^2}\ \alpha d\kern2em \mathrm{for}\kern0.5em d\ll {w}_{\mathrm{z}} $$

where *α* is the Raman scattering (or generation) coefficient per unit length, and 2*w*_Z_ is the full half width (FWHM) of the Lorentz distribution curve, a parameter that describes the depth resolution of the optical setup. The profile of Eq. (8) depends on the pinhole diameter *D*, the numerical aperture NA of the objective, and its magnification factor *M*. An empirical relation was developed by Wilson [36].

9 $$ {w}_{\mathrm{z}}=0.33\lambda \sqrt{\frac{1+{AU}^2}{n-\sqrt{n^2-{\mathrm{NA}}^2}}\kern0.5em }\ \mathrm{where}\  AU=D\cdotp \mathrm{NA}/\left(0.61\lambda \cdotp M\right) $$

The depth profiles of layers with arbitrary thickness *d* > 0 are accessible by summing up all contributions from the front layer surface *z*_S_ to the rear layer surface *z*_S_ + *d*. The result depends on the position of *z*_S_ versus *z*_0_. An analytical solution is obtained with Eq. (8) as integrand

10 $$ {X}_{\mathrm{R}}\left({z}_0,{w}_{\mathrm{z}},d\right)\sim \left(\arctan \left(\frac{d-{z}_0}{w_{\mathrm{z}}}\right)+\arctan \left(\frac{z_0}{w_{\mathrm{z}}}\right)\right)\upalpha {w}_{\mathrm{Z}} $$

where the zero point of *z* is chosen at *z*_S_. All *z*_0_- and associated mobile stage *z*_M_-positions are then counted negative in front of the surface (pre-focal range), and positive inside the layer.

#### Evaluation procedures

**Fig. 4:**
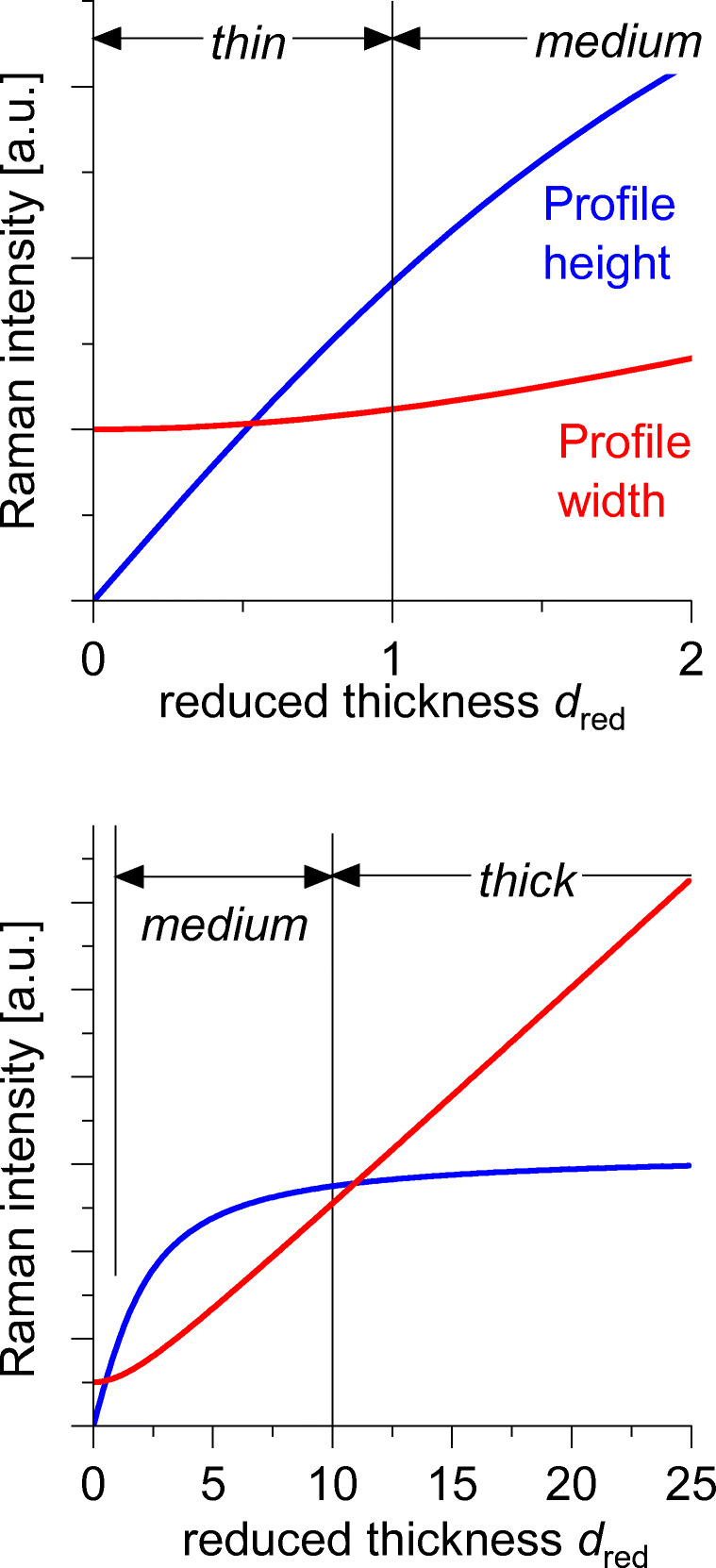
Depth profile widths (FWHM, red curve) and depth profile maximum heights (blue curve, arbitrary ordinate) as function of the reduced layer thickness *d*/*w*_z_. Top: thin and medium layer regime. Bottom: medium and thick layer regime

According to Eq. (10) and Fig. 4, the systems under investigation can be classified into

- *thin layers* with the reduced thickness *d*_red_ = *d / w*_Z_ < 1, where the signal height increases linearly with the layer thickness *X*_R_ ~ *d* and the signal *z*-shape remains Lorentzian with constant width FWHM = 2*w*_Z_.
- *thick layers* with the reduced thickness *d*_red_ > 10, where the signal height maximum is almost constant *X*_R,max_ ≈ const and the signal *z*-width increases linearly with the layer thickness FWHM = *d*. In addition, the normalized pre-focal slope of the *X*(*z*)-curve enables the determination of *w*_Z_.
- *medium layers* with 1 < *d*_red_ < 10, where both *X*_R,max_ and FWHM vary with *d*_red_. The two variables *w*_Z_ and *d* are accessible from least squares fits of the experimental *z*-scans. Alternatively, *w*_Z_ is taken from *thin* or *thick* layers so that *d* can be directly determined from the *z*-trace.

Equation (10) is designed primarily for index-match but, as will be seen in the “Experimental results” section, can be used for thin layers or small apertures also in the case of mismatch. A more general description of the systems is obtainable by numerical integration of Eq. (1) with the help of Eqs. (2–7). Input parameters are *R*_L_, *z*_0_, *n*, and the variable position of the layer surface *z*_S_ during the *z*-scan. The potential emitters G(*r*, *z*) are excited from *z*_S_ to *z*_S_ + *d*_real_ under different internal polar angles and therefore different intensities according to Eqs. (5, 6, and 7). The excitation intensities include the influence of focal aberration with increasing polar angle. The emitted intensities follow the field of vision (vide supra), under consideration of angular focal aberration, and refraction or total internal reflection at *z*_S_. The calculated *X*_R_(*z*)-traces are fitted to the experimental results by variation of *d*_num_, *w*_P_, and *ff*. The layer thicknesses are labeled as detailed in Table 3. Inclusion of *w*_0_ as the fourth variable makes fitting rather uncomfortable. The value of *w*_0_ was therefore taken with little loss of accuracy from the radius of the Airy-disk for given NA and magnification of the objective. The focused spot size was also experimentally detected by mirror reflection to *w*_0_ = 0.345 μm (100×/0.90) [21] and to *w*_0_ = 0.32 μm (100×/0.85) [28].

## Experimental results

### The thin layer regime

The geometrical or optical relevant layer thickness is lower than the effective depth resolution 2*w*_z_ of the micro-spectrometer. The optical thicknesses of e.g. graphene or silicon follow this criterion. According to the Si-absorption coefficients at the excitation (*k*_532_ = 7.7·10^3^ cm^−1^) and Raman emission wavelengths (*k*_547_ = 6.9·10^3^ cm^−1^), and the refractive index *n* = 4.1 [37], the optical sampling depth *d*_opt_ = (*k*_532_ + *k*_547_)^−1^*n*^−1^ = 170 nm is distinctly lower than the experimental depth profiles measured with the three objectives. The profiles of NA = 0.4 and NA = 0.7 exactly follow the Lorentzian of Eq. (8), whereas NA = 0.9 slightly broadens in the central region by convolution with the sampling depth of silicon. Table 4 collects the experimental *w*_z_-values of the device. The table also presents the model calculations according to Eq. (10) which are in good agreement with the experiments.

**Table 4:** Some calculated and experimentally fitted geometric parameters of micro-Raman depth profiling

**Microscope objective**	**20×/0.4**	**50×/0.7**	**100×/0.90**
**Effective depth resolution*w***_**Z**_**/μm**			
**Calculated**			
Eqs. (8 and 10) *n* = 1.59 *D* = 25 μm	*w*_z_ = 2.8	0.8	0.33
**Experimental**			
Thin layer (silicon)	2.85	0.76	0.35
Thick layer (polystyrene) pre-focal profiles, Fig. 7, Eq. (10)	2.7	0.65–0.7	0.27
Slope maximum at *z*_S_ = *z*_0_, Eq. (11)	2.53	0.81	0.31
Overall profiles, Eq. (10), Fig. 8	2.65	n.a.	n.a.
**Effective pinhole image radius*w***_**p**_**/μm**			
Mechanical pinhole diameter			
*D* = 25 μm	*w*_P_ = 1.35–1.4	0.85	0.35
*D* = 200 μm [23] Fig. 9			1.6
*D* = 400 μm [23] Fig. 9			2.5
**Waist radius of excitation*w***_**0**_**/μm**	*w*_0_ = 1.0–1.1	0.7	0.35
**Objective fill factor of excitation*ff***	*ff* < 0.45	0.45–0.55	0.75–0.85

### Thin to medium layers

**Fig. 5:**
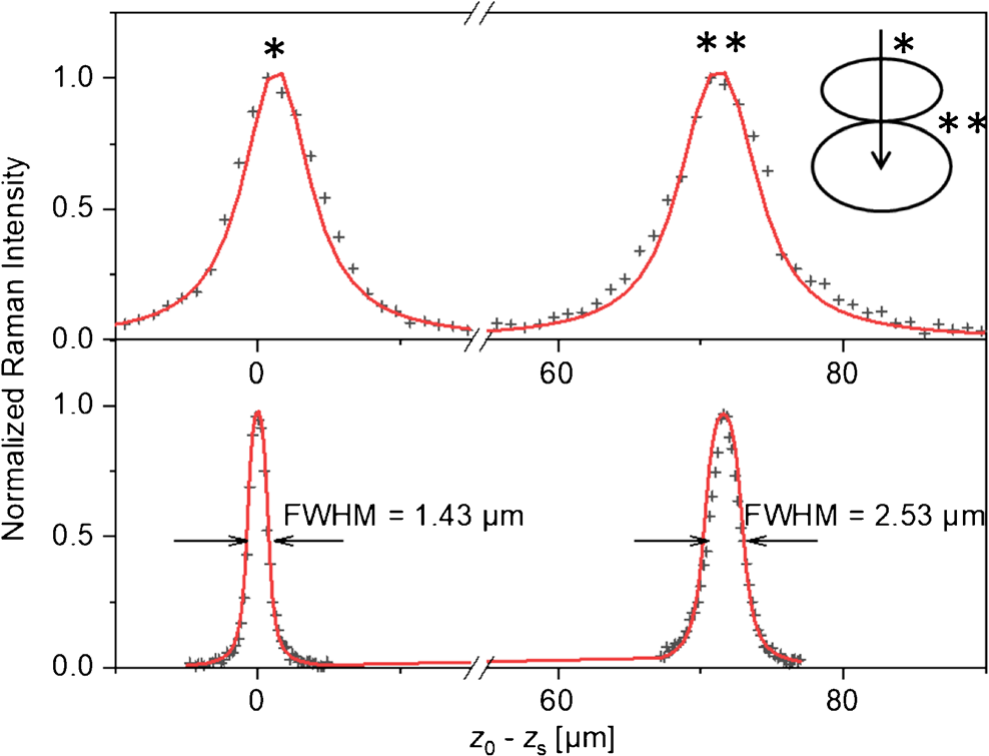
Raman depth profiles of thin bubble membranes in polystyrene foam. Top: 20×/0.4 objective, bottom: 100×/0.9 objective. The distance between the two maxima for both profiles is approximately 72 μm, corresponding to the size of the first bubble. All intensity maxima are normalized to unity. Crosses: experimental profiles with indication of FWHM; lines: analytical fit with Eq. (10). Inset: schematic representation of the measurement setup, with the excitation beam (arrow) approaching the first bubble surface (*) and then the contact surface (**) between the first and the second bubble

Styrodur is a rigid foam variant of polystyrene with, in our sample, a specific density of 0.06 g cm^−3^. The material is formed from densely packed, gas-filled bubbles of irregular shape and size of ca. 120–220 μm in the long direction and 60–100 μm in the short direction. To the naked eye, the material appears turbid with an effective elastic scattering coefficient of *σ* = 35 cm^−1^ that is almost independent of the wavelength [11]. Microscopically, however, the bubbles are fairly transparent, i.e., free of distortion by multiple scattering from region D in Fig. 1. We analyzed the thickness of the bubble walls via Raman *z*-scans focused at the outer material surface and in the interior. Figure 5 presents typical *z*-traces and their curve fits. As in the case of silicon, the trace with NA = 0.4 (*M* = 20×) falls into the *thin layer* regime and reflects the depth resolution of the objective, whereas the trace with NA = 0.9 (*M* = 100×) has to be treated as layer of *medium thickness* yielding the outer wall thickness *d*_exp_ = 1.4 μm and, in a depth of *z* = 74 μm, the inner thickness *d*_exp_ = 2.7 μm. The latter we tentatively interpret as intimate contact of two adjacent bubbles (see inset Fig. 5, top right).

**Fig. 6:**
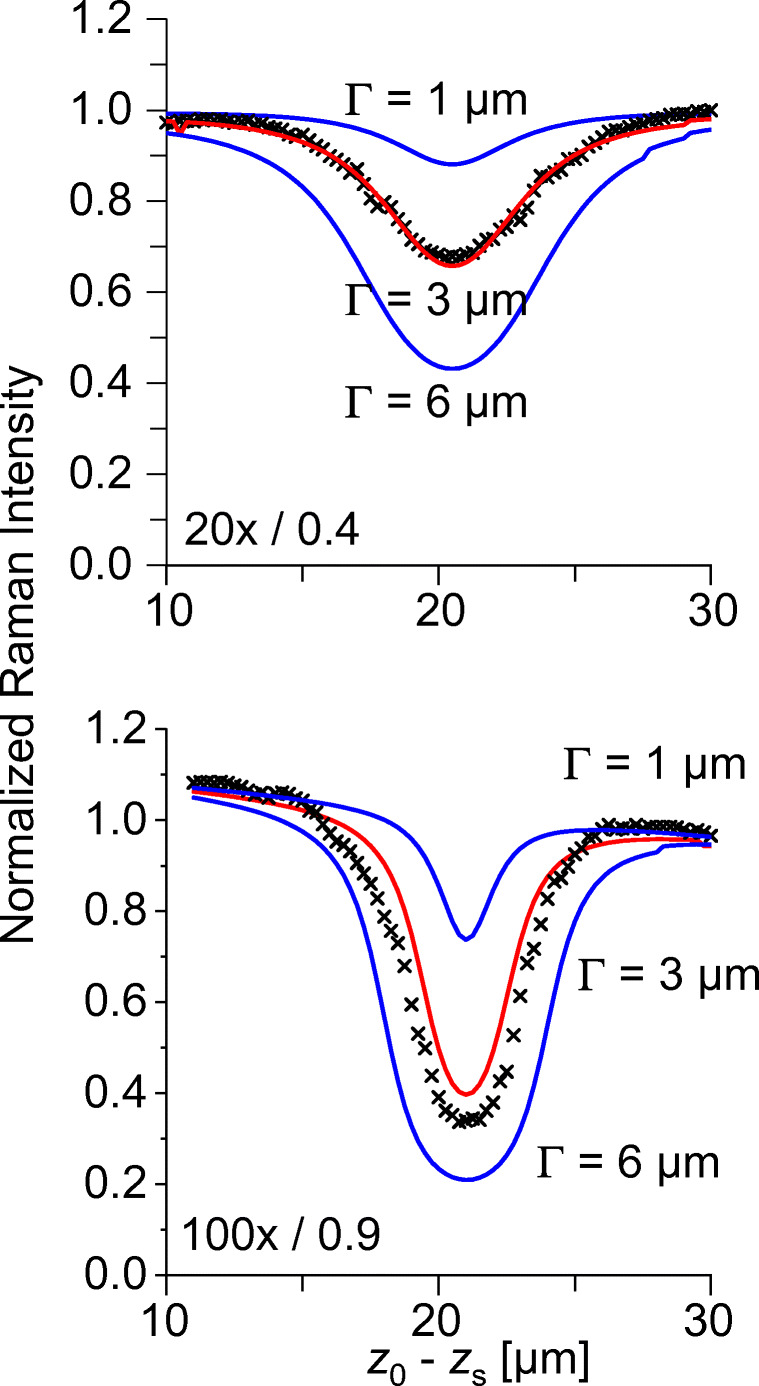
Raman depth profiles of two PE layers which are separated by a thin EVOH layer, within a packaging film for the food industry. Top: 20×/0.4. Bottom: 50×/0.7. Crosses: experimental profiles indicating an optical gap *Γ* of approx. 3 μm, lines: analytical solutions of Eq. (10) calculated for the gap widths *Γ* = 1 μm, 3 μm, and 6 μm, respectively

If the layer is below the depth resolution of the device, its thickness can be determined from the Raman intensity. In this case, an intensity reference is required in analogy to absorption spectroscopy. The following example uses as internal reference the Raman active matrix in which a thin interlayer is embedded that forms a gap in the matrix. The gap produces a sink in the Raman z-scan whose depth is a function of the gap width. Figure 6 presents experimental and calculated results in a polyethylene matrix with a *Γ*_real_ = 5-μm polyalcohol interlayer, located 30 μm below the matrix surface. The interlayer causes an intensity attenuation of 30% (20×/0.4) and 65% (50×/0.7), respectively, and the *z*-traces yield with Eq. (10) an optical gap width of *Γ*_exp_ = 3–3.5 μm. The figure also shows clearly that intra-material gap widths of *Γ* < 1 μm can be assigned with low objective aperture, i.e., without intensity loss and shape distortion in deep sample regions, using this method.

### Thick layers

#### *The pre-focal range z*_S_ > *z*_0_

**Fig. 7:**
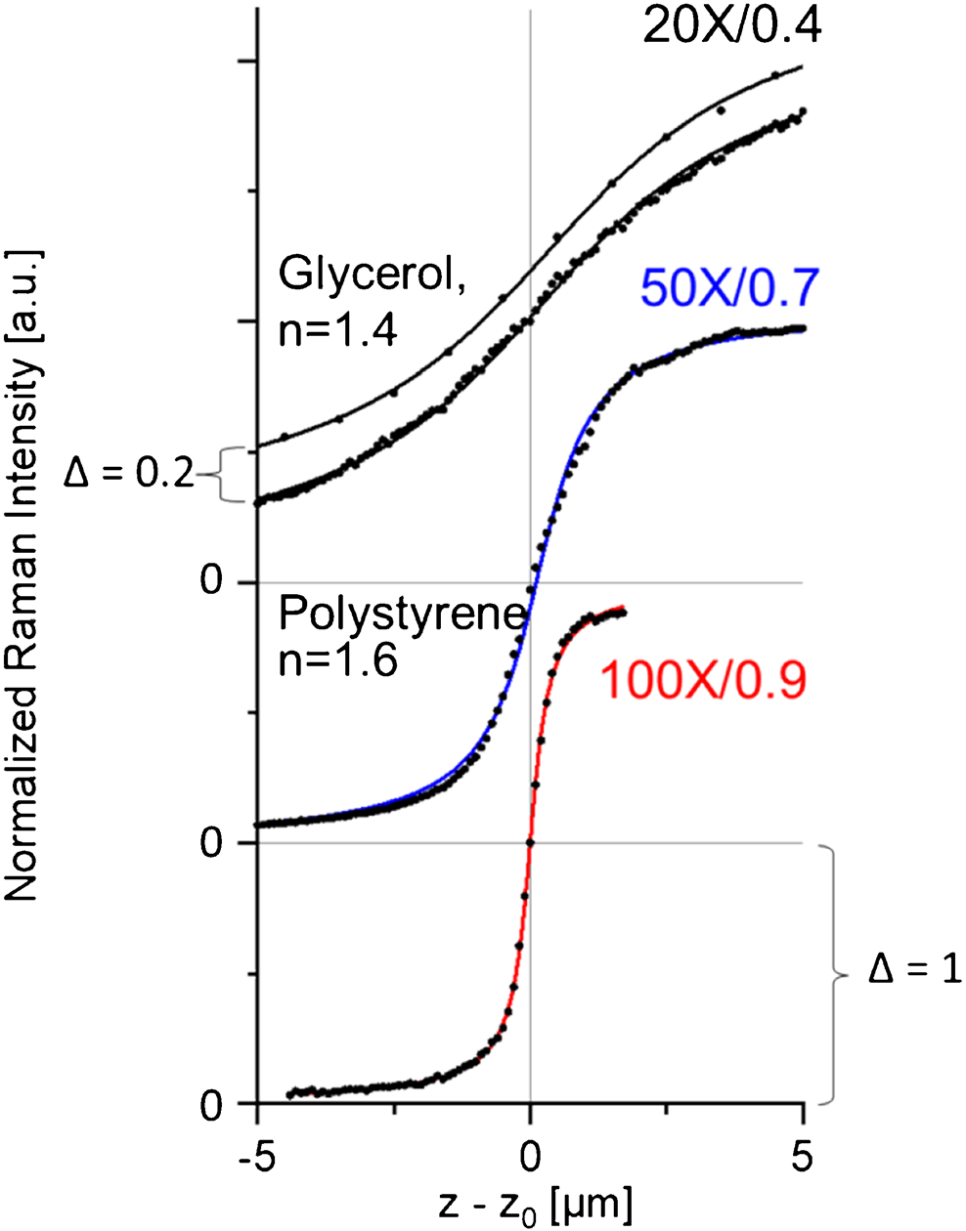
Raman *z*-scans of a thick transparent polystyrene layer, measured in the pre-focal and beginning in-focal range with three different objectives. The experimental data (dots) are normalized at the sample surface to unity. The solid lines represent depth fits using Eq. (10) with *w*_z_-values summarized in Table 4. For better visualization, the data are displayed with offset Δ = 1. The *z*-scan of glycerol (on top with offset Δ = 0.2) shows that the normalized curve progression is independent of the refractive index *n*

Due to the lack of angular aberration in *z*_0_ and the applicability of Eq. (4) in its small-angle approximation, this range can be treated with relatively simple mathematics. Figure 7 shows some of our experimental *X*_R_(*z*)-profiles.

The data are normalized to the values at *z*_S_ = *z*_0_. This procedure eliminates the linear dependence on the Raman generation coefficient as well as the reciprocal dependence on the refractive index, and highlights the influence of the pinhole magnification and objective aperture.

The full lines are fitted with Eq. (10) or with our numerical procedure without revealing significant deviations from the experiments. Slight deviations become recognizable only if the overall fit is compared to the slope maximum of Eq. 10

11 $$ \frac{\mathrm{d}{X}_{\mathrm{R}}(0)}{\mathrm{d}z}=\frac{2}{{\pi w}_{\mathrm{z}}}\ \mathrm{with}\ {X}_{\mathrm{R}}(0)=1 $$

Table 4 summarizes the fitted geometric parameters of the device. It should be noted that the values of the thick layers come very close to the thin layer limit which supports the physical relevance of our data.

#### The *in-focal range*

**Fig. 8:**
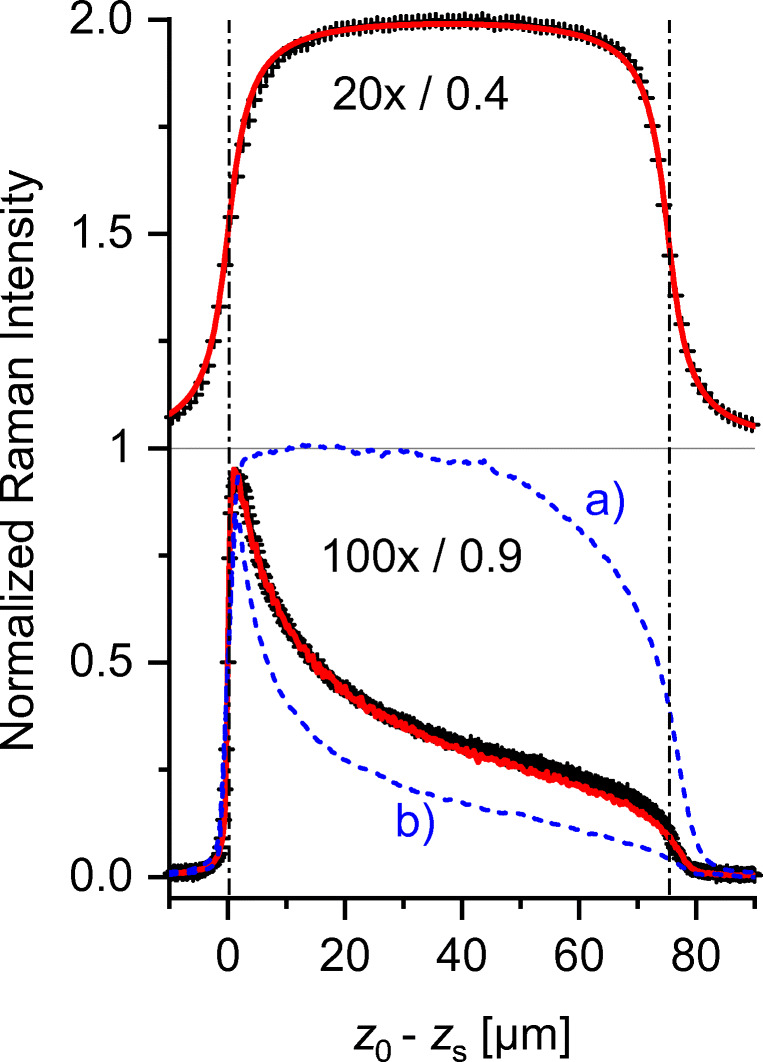
Raman depth profiles of transparent polystyrene layers measured with different optical setups 20×/0.4 (top) and 100×/0.9 (bottom). Black crosses: experimental data; red lines: simulations of the experiments; blue lines: numerical simulations for fill factor (a)*ff* = 0.001 and (b)*ff* = 2^½^

Figure 8 presents the complete *z*-scans of a transparent poly-styrene layer with real thickness *d*_real_ = 124 ± 0.5 μm. The small-aperture trace can be perfectly fitted over the whole depth range with the analytical model of Eq. (10) yielding *w*_Z_ = 2.65 μm and the optical thickness *d*_opt_ = 74.8 μm, which is equal to the experimental thickness *d*_exp_ = 74,7_5_ μm obtained from the 1st derivative extremes of the experimental trace. As described in a series of publications [15, 16, 29, 38], the large-aperture traces deviate significantly from the symmetry of Eq. (10). Our numerical fits, with the pre-focal*w*_P_-values as base for FV, require the fill factor *ff* of the excitation beam through the objective as important scaling variable (see Eqs. (5 and 6)). A reasonable fit of the experimental 100×/0.9 – trace in Fig. 8 is obtained with *ff* = 0.85, whereas the curve with *ff* = 1 is clearly away from reality. The other fitting parameters *w*_p_ = *w*_0_ = 0.35 μm were inserted as limit given by the Airy diffraction disk of *w*_Airy_ = 0.61λ_0.532_/NA = 0.36 μm. In total, the calculated overall *z*-scan profiles are in excellent agreement with the experimental curves. Systematic deviations are found only close to the rear sample side where improvement could be achieved by addition of the 5% internal back reflection of the laser waist at the rear sample surface.

**Fig. 9:**
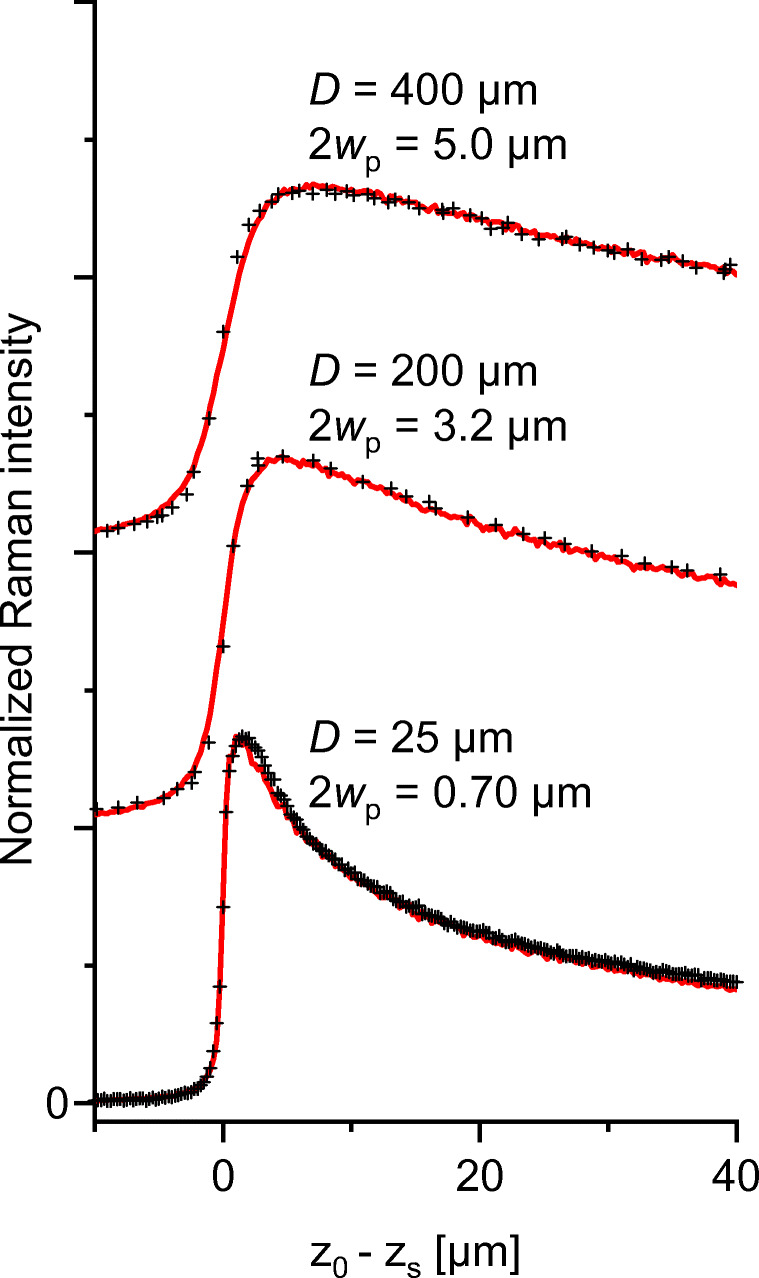
Raman *z*-scans of a thick transparent PS layer measured (black crosses) with NA = 0.9 (*M* = 100×) objective and different pinhole diameters *D*. Experimental data for *D* = 200 μm and 400 μm from Esposito et al. [23] and 25 μm from authors’ measurements. Curve fits (red, this paper) yield the effective pinhole image diameters 2*w*_P_ in the focal plane

Larger pinhole diameters *D* yield larger image diameters with lower depth resolution but less intensity loss in the deep regions. Figure 9 describes this behavior with the help of experimental literature data [23], evaluated with the formalism of the present work. The fitted image diameters are always somewhat widened by diffraction against the geometric magnification ratio 2*w*_P_ > *D*/*M*.

### Bi-layer stack

The central part of this work quantifies the depth-dependent signal intensities and profile shapes in composite layers with known thicknesses and depth positions of the individual components. Application-oriented examples were found in high-tech packaging materials where different polymers are stacked in a multilayer film in order to achieve mechanical fracture strength and low gas permeability. We measured several samples with promising results but limited data accuracy because of local thickness variations in the range of 1–2 μm, and residual elastic scattering at the internal phase boundaries. We achieved the best results with an adhesive tape that was stacked to form a two-component series ABABABAB, where A = highly transparent polypropylene (PP) and B = adhesive polymethacrylate (PMA) resin. The thickness of the tape AB was determined optically by Raman *x*-scans along the flat face of the intact tape roll, and mechanically by the thickness of the complete roll with known tape length. Both methods yielded an equal thickness of *d*_AB,real_ = 40.2 ± 0.3 μm.

**Fig. 10:**
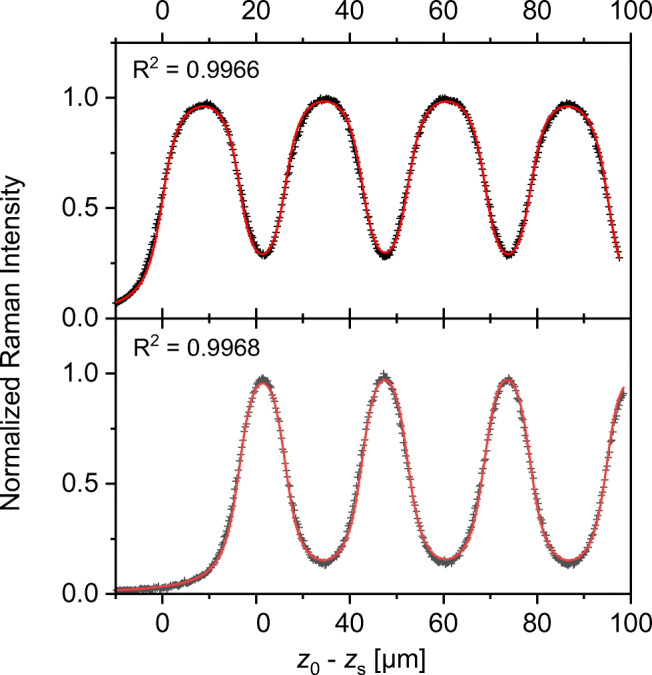
20×/0.4 optical setup. Raman depth profiles of quadruple stack of polypropylene (PP)/acrylate resin adhesive tape. Top: PP profile. Bottom: acrylate profile. Black crosses: experimental data. Red lines: analytical fits with Eq. (10), correlation coefficients as displayed and fit variables as in Table 3

**Fig. 11:**
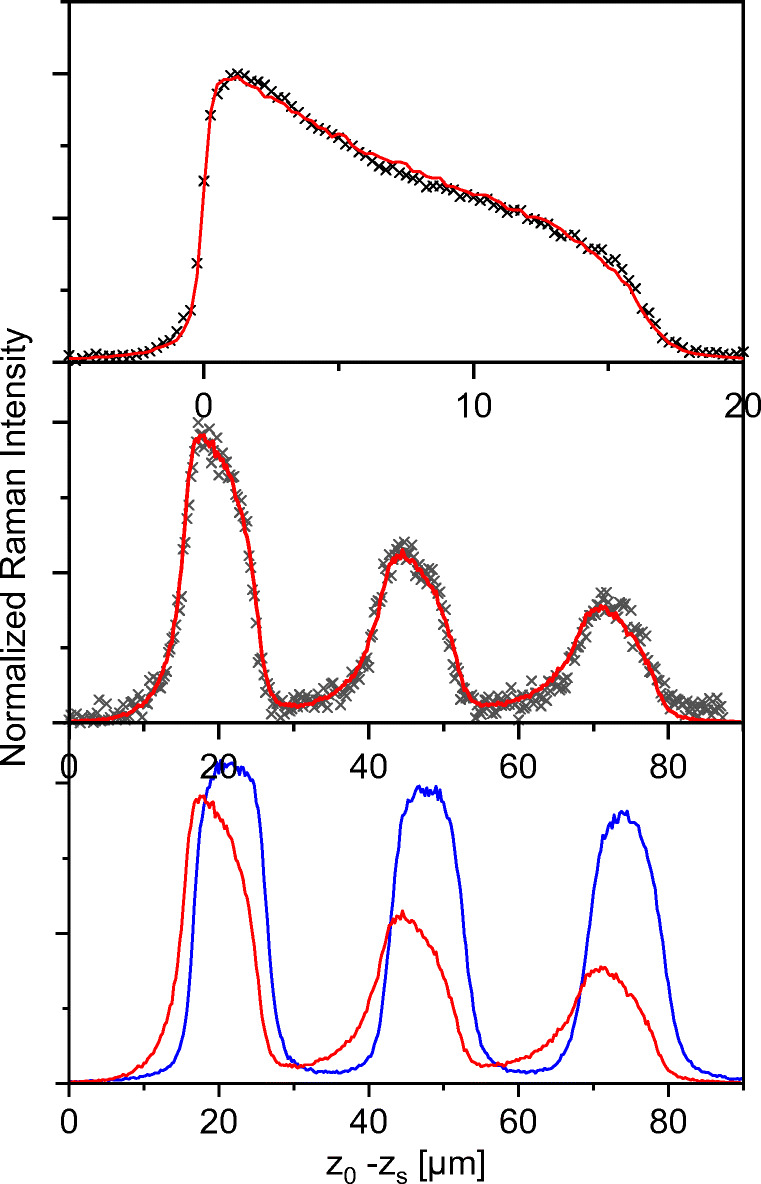
Optical setup 100×/0.9. Raman depth profiles of the same sample as in Fig. 10. Top: enlarged view of the first PP layer. Center: acrylate layer. Black crosses: experimental data with 100×/0.9 optical setup. Red lines: corresponding numerical fits, by using the parameters according to Table 4. Bottom: Numerical simulations of the acrylate profiles using the two optical setups with 100×/0.9 (red line) and 100×/0.4 (blue line)

Figures 10 and 11 show *z*-scans of the stack evaluated at the C=O Raman frequency Δ*ν* = 1730 cm^−1^ which is present only in the B-component, and at the skeleton Raman frequency Δ*ν* = 399 cm^−1^ which is present only in the A-component. The traces of the two components are well separable with all objectives.

The *z*-profiles with NA = 0.4 (*M* = 20×) (Fig. 10) are highly symmetric with constant maxima heights, minima depths, and period widths over the whole stack. Hence, the low-aperture device avoids information loss in the deep regions of composite layers, at least for *medium* or *thick* components.

Due to the agreement between the experimental and fitted curves, reliable data about the virtual positions and dimensions of the layer components were obtained, as presented in Table 3. The comparison with the 1st derivatives shows clearly that both methods deliver the same results, which supports the quantitative applicability of Eq. (10).

Table 3 presents fitted results obtained with the formal procedure of Eq. (10) and with the numerical geometric evaluation. In the separate fits of A and B (Fig. 10), the gaps between A correspond exactly to the thickness of B and vice versa*.* The global fit comes off a little worse because the two traces are shifted against each other by Δ*z* = 1.6 μm due to chromatic aberration between the two detection wavelengths.

In contrast to the latter, the *z*-scans with NA = 0.9 (*M* = 100×) (Fig. 11) are highly asymmetric with intensity loss and profile distortion in the deep layer regions. The numerical fit of the first A-trace reproduces with *d*_num,A_ = 26.2 μm the experimental curve very well. The thickness of the B-component is then obtained as *d*_B_ = *d*_AB_–*d*_A_ = 40.2 – 26.2 = 14.0 μm.

This value was used to simulate, without further corrections, the B-trace over the whole stack. As can be seen in the middle part of Fig. 11, the agreement with the experiment is extremely good, not only in the signal widths and heights, but also in the asymmetric tailing that arises from the forward component of the FV (see Fig. 2) which increases with the depth position. The lower part of Fig. 11 offers a suggestion how to reduce tailing and intensity loss under maintenance of high depth resolution. The theoretical curve uses an additional iris in the microscope tube in order to truncate wide emission angles.

## Discussion and conclusion

**Fig. 12:**
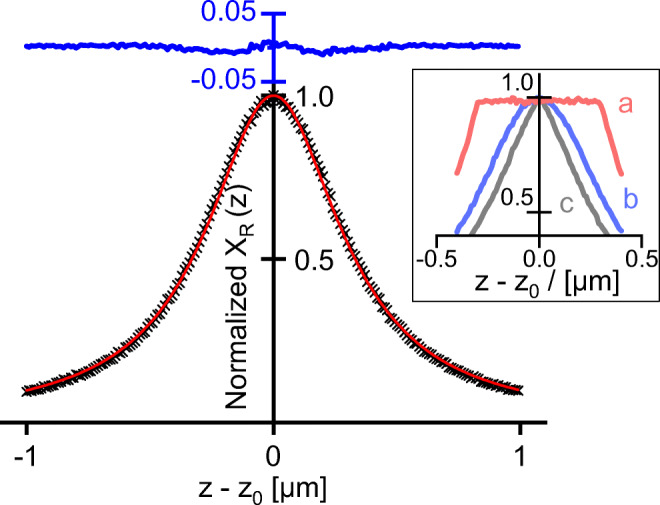
Modeled Raman depth profiles of an ultra-thin layer (*d* ➔ 0) with a signal maximum at *z*_0_ = 0, *z*_F_ = 1000 μm. Black crosses: random walk simulation for lens radius *R*_L_ = 1000 μm, Gaussian irradiation through full lens with irradiation waist radius *w*_0_ = 0.3 μm, pinhole at *z*_p_ = 1000 μm with pinhole radius *w*_P_ = 0.3 μm. Red line: best Lorentzian fit of Eq. (10) with *w*_eff_ = 0.33 μm. Blue line: the difference between the two results. Inset: depth profiles for (a)*δ*-irradiation, (b) Gaussian irradiation, (c) homogeneous irradiation through the pinhole area

The experimental data of this work were collected from transparent amorphous layers with very low to negligible elastic scattering power. Under these conditions, it is possible to test simple optical models that describe the depth response of confocal microscopic Raman or fluorescence experiments. The model with the analytical solution of Eq. (10) requires focused laser-irradiation with Gaussian radial intensity profile and yields the formal depth resolution FWHM = 2*w*_Z_ of the device, the virtual depth center *z*_vir_ of the detected Raman signal, as well as the virtual thickness *d*_opt_ of the emitting layer. The geometric model with numerical solution also requires focused irradiation, but can freely choose the radial intensity profile. Figure 12 compares calculated Raman depth profiles for homogeneous, Gaussian, and δ-irradiation.

### The real depth position of the Raman emitter in a refractive medium

Knowledge of the refractive index is the first prerequisite for determining the emitter’s depth position. As a rule of thumb, the virtual position, as defined for thin interlayers by the experimental emission maximum in the *z*-scan, has to be multiplied by ***n*** in order to get the real depth. A higher accuracy is achieved if additionally, the focal aberration is considered as a second correction factor. Table 5 presents the ratio of the *real* to the *n · virtual* depth position of a thin object layer as calculated from the emission maximum for different apertures and refractive indexes and shows clearly that this additional correction depends strongly on the refractive index of the matrix and the objective aperture. Close to the sample surface, the correction factor increases with depth, but the factor saturates in deeper regions to the values as given in Table 5*.*

**Table 5:** Ratio of the *real* to the *n · virtual* depth position of a thin object layer, details in text

	$$ \frac{\left({\boldsymbol{z}}_{\mathbf{real}}-{\boldsymbol{z}}_{\mathbf{S}}\right)}{\boldsymbol{n}\left({\boldsymbol{z}}_{\mathbf{vir}}-{\boldsymbol{z}}_{\mathbf{S}}\right)} $$	
	***n*** **= 1.5**	***n*** **= 2**
**NA 0.2**	1.00–1.005	1.00–1.01
**NA 0.4**	1.03–1.04	1.06–1.07
**NA 0.9**	1.07–1.08	1.11–1.12

In the *thick interlayer* range, it is advisable not to define the maximum, but the *z*-boundaries of the interlayer as a position marker that can be obtained by e.g. the 1st derivative extremes or the half-height values of the *z*-trace. As can be extracted from the experimental data of the stacked adhesive tape, the shape of the trace and the correction factor of Table 5 remain, with NA = 0.4 (*M* = 20×), constant for all depth positions of the components in the stack. Thus, the real positions can be determined with an uncertainty of ±0.25 μm. One might expect that the accuracy should be even better with NA = 0.9 (*M* = 100×). However, the aberration correction depends in this case on the position of the interlayer in the stack. The uppermost A-component is experimentally determined from the 1st derivative as *d*_exp,A_ = 16.25 μm. The experimental thickness increases in the deep region to *d*_exp,A_ = 17.3 μm because of the tailing of the lower *z*-boundary, whereas the result with NA = 0.4 (*M* = 20×) stays constant with *d*_exp,A_ = 16.5 ± 0.25 μm over the whole stack. A very similar behavior is observed for the experimental thickness of the complete AB-tape as function of its stack position, as can be extracted from Table 3.

**Table 3:** Evaluation of the polystyrene *z*-scans of Fig. 8, and adhesive tape of Fig. 10 and Fig. 11; all length data in μm

**Evaluation procedure**					
**Layer system** **Physical thickness, μm**		**Numerical**	**Analytical, Eq.****10**	**Experimental,** **from 1st derivatives**	
		***d***_**num**_**, μm**	***d***_**an**_**, μm**	***d***_**exp**_**, μm**	***d***_**real**_**/*n*·*d***_**exp**_
**Polystyrene** ***d***_**real**_ **= 124 ± 0.5 μm**					
	**NA = 0.4** **NA = 0.9**	123.5 ^(a)^ 126 ^(b)^	74.8 n.a.	74.7_5_ 75.7_5_	1.04_3_ 1.03
Quadruple stack of **adhesive tape AB** ***d***_**real**_ **= 4 x (40.2 ± 0.3) μm**					
	**NA = 0.4**				
	**AB-layer** average ^(^*^)^ sum of #1 to #3			26.3 ± 0.5 ^(^*^)^ 79.5	
	**A-layer** gap between A’s		16.4_9_^(^**^)^ 9.6_1_	16.5	1.03
	**B-layer** gap between B’s	14.5 ^(a)^ 26.2_5_^(a)^	9.6_4_ 16.5_1_	9.7_5_	
	**NA = 0.9**				
	**AB-layer #1** **#2** **#3** sum of #1 to #3			25.8 26.2 26.4 78.2	
	**A-layer #1**	26.2 ^(b)^		16.2_5_	1.04_7_
	**B-layer #1**	14.0 ^(b)^		9.2_5_	

^(a)^Results with *w*_0_ = 0.8 μm, *ff* = 0.4 *w*_P_ = 1.34 μm

^(b)^Results with *w*_0_ = 0.35 μm, *ff* = 0.85 *w*_p_ = 0.35 μm

*(^)^No trend with depth position

**^()^Results of least square fits. The quality of the fitting given as correlation coefficient is displayed in Fig. 10

If deviations in the range of ±1 μm are accepted, the thicknesses extracted from NA = 0.4 and NA = 0.9 are substantially equal. If sub-micrometer precision is required, the two apertures yield slightly different results in the sense that the first AB-component of the adhesive tape stack is optically more compressed with NA = 0.9 than with NA = 0.4 (see the experimental data). This result is a consequence of RIM that shifts the origin of large emission angles virtually stronger to the irradiated surface than small ones. The effect is partly compensated in the deeper AB-components by the forward tailing of the FV (Figs. 2 and 3) which increases the optical thickness with NA = 0.9 but not with NA = 0.4. In total, the effect is small and only a by-product against the main issue of Table 3 to demonstrate that analytical methods are able to well quantify deeply located layer components, provided the system is free of elastic scattering.

### The fill factor of excitation

Traditionally, the fill factor *ff* of a given area or volume is normalized to unity for complete homogeneous occupation. Upon collimated TEM_00_ irradiation of the lens area, this type of normalization requires wide beam expansion or space filtering with unrealistic intensity loss for the detection of the weak Raman signals. On the other hand, a wide beam produces a small spot radius *w*_0_ with high spatial resolution but, unfortunately, also with strong depth intensity loss in the case of refractive index mismatch. In a reasonable compromise, the beam is expanded only as wide as necessary so that the intensity is reduced at the lens boundary to the ratio (fraction)*e*^−*n*^ relative to the center. According to the definitions in Eqs. (5) and (6), the ratio *e*^−1^ corresponds to *ff* = 2^1/2^, *e*^−2^ to *ff* = 1, *e*^−4^ to *ff* = 2^–1/2^, etc. The experimental *ff*-data of Table 4 are obtained from the best fits of the *z*-scans. At the moment, it is not known how much the data are of physical reality and how much they are subject to the simple model assumptions, especially to the neglect of the electromagnetic wave character of irradiation. A photometric determination of the expanded laser cross section will help. As can be seen from Fig. 8, the influence of *ff* on the depth profiles is important. Since the diameters of the objectives, but not of the excitation beam increase with falling NA (see Table 2), the *ff*-value of NA = 0.4 becomes low, a prerequisite for the symmetrical depth profiles measured with this objective.

### The spatial confinement of the detected signal

The spatial confinement of the detected signal offers a measure for the local resolution of CRM. With normalization of the detection field FD (photons × time^−1^) to its maximum value, the integrated Eq. (1) delivers the detection volume *V*_c_, often also named *confocal volume*. Unfortunately, *V*_c_ cannot be described by rigid boundaries since the shape of FD varies strongly with *r* and *z*. Nevertheless, the volume is sometimes symbolized by a rotational ellipsoid (rugby ball). A somewhat more realistic shape can be borrowed from an acoustic horn or from a Yo-Yo, since the detectable photon distribution widens with the distance from *z*_0_, but also loses density. In a logarithmic contour plot, the widening becomes clearly visible [21]. The region close to *z*_0_ offers a simple description of Δ*V*_c_ = *a* · π *w*_0_^2^ Δ*z*, where Δ*z* < *w*_0_ is the thickness of a thin layer around *z*_0_, and *a* takes into account the radial profile of FD. A Gaussian excitation profile yields *a* ≈ 0.5 with partial truncation through the pinhole if *w*_P_ is not significantly wider than *w*_0_. The numerical evaluation over a thick layer yields for index match *V*_c_ (100×/0.9) = 0.3 μm^3^ and *V*_c_(20×/0.4) = 12 μm^3^, respectively. Index mismatch increases both volumes by more than a factor of *n.* In addition, the axial extension of *V*_c_ (100×/0.9) increases significantly into the +z-direction with the depth position, whereas *V*_c_(20×/0.4) stays constant.

Therefore, the advantage of high versus low optical resolution is increasingly lost in the deep hidden depth.
